# Early oral nutritional supplementation and postoperative recovery after hip arthroplasty in malnourished older adults with femoral neck fracture

**DOI:** 10.3389/fnut.2026.1840514

**Published:** 2026-07-22

**Authors:** Jia-Hui Xu, Jing-Xin Niu, Zhen Wang, Chun-Feng Liu

**Affiliations:** 1 Department of Orthopedics, Suzhou Kowloon Hospital, Shanghai Jiao Tong University School of Medicine, Suzhou, Jiangsu, China; 2 Department of International Medical Center, Suzhou Kowloon Hospital, Shanghai Jiao Tong University School of Medicine, Suzhou, Jiangsu, China

**Keywords:** early enteral nutrition, femoral neck fracture, hip arthroplasty, malnutrition, nutritional risk screening 2002, older adults, postoperative recovery, pulmonary infection

## Abstract

**Background:**

Older patients with malnutrition who undergo hip arthroplasty for femoral neck fracture are at high risk of delayed postoperative recovery. This retrospective study aimed to examine the association of early oral nutritional supplementation (ONS) with postoperative nutrition-related laboratory indicators, recovery outcomes, and in-hospital complications in this population.

**Methods:**

We retrospectively analyzed consecutive patients aged 60 years or older with malnutrition who underwent hemiarthroplasty or total hip arthroplasty for femoral neck fracture between January 2022 and December 2025. Patients were classified into an early ONS group if structured ONS was initiated within 24 h after surgery and into a control group otherwise. Primary outcomes included postoperative changes in serum albumin, prealbumin, hemoglobin, total protein, lymphocyte count, length of hospital stay, time to first ambulation, and time to first flatus. Secondary outcomes were postoperative complications during the index hospitalization.

**Results:**

Among 294 included patients, 118 received early ONS and 176 served as controls. At discharge, the early ONS group showed more favorable laboratory profiles, including higher serum albumin [*β* = 1.42, 95% confidence interval (CI): 0.66 to 2.18; *p* < 0.001], prealbumin (*β* = 13.58, 95% CI: 5.92 to 21.24; *p* < 0.001), total protein (*β* = 1.76, 95% CI: 0.54 to 2.98; *p* = 0.005), and lymphocyte count (β = 0.11, 95% CI: 0.04 to 0.18; *p* = 0.002). Early ONS was also associated with shorter time to first ambulation (*β* = −5.38 h, 95% CI: −7.56 to −3.20; *p* < 0.001), shorter time to first flatus (*β* = −6.84 h, 95% CI: −9.53 to −4.15; *p* < 0.001), and shorter hospital stay (*β* = −1.29 days, 95% CI: −1.98 to −0.60; *p* < 0.001). Lower odds of pulmonary infection were observed (odds ratio = 0.46, 95% CI: 0.16 to 0.93; *p* = 0.041), although this finding should be interpreted cautiously because of the limited number of events.

**Conclusion:**

Early ONS after hip arthroplasty in older patients with malnutrition and femoral neck fracture was associated with more favorable short-term nutrition-related laboratory profiles and faster postoperative recovery. The association with pulmonary infection warrants cautious interpretation and further validation in prospective studies.

## 1 Introduction

Femoral neck fracture is a major cause of disability, loss of independence, and excess perioperative morbidity in older adults, and hip arthroplasty remains a standard surgical strategy for displaced fractures in this population (1, 2). Despite advances in anesthesia, implant selection, and orthogeriatric care, postoperative recovery in elderly patients is frequently complicated by frailty, multimorbidity, impaired mobility, inflammatory stress, and limited physiological reserve (3, 4). Recent literature has increasingly emphasized that nutritional status is a central determinant of outcome after hip fracture surgery, rather than a secondary background characteristic (5–7). Poor nutritional status has been associated with delayed rehabilitation, prolonged hospitalization, higher complication rates, and worse survival after hip fracture (5). Malnutrition is particularly relevant in older patients undergoing surgery for femoral neck fracture because the catabolic effects of acute trauma are superimposed on pre-existing sarcopenia, anorexia of aging, chronic disease burden, and reduced dietary intake (8). In this setting, perioperative nutritional depletion may aggravate muscle loss, impair immune competence, delay wound healing, and reduce tolerance to mobilization and rehabilitation (9). Contemporary evidence indicates that nutritional indices and screening tools are prognostically informative in elderly patients with hip fracture, with impaired nutritional status being associated with mortality, postoperative delirium, functional dependence, and other adverse outcomes (10–12).

Postoperative nutritional support has therefore become an area of growing interest in geriatric fracture care. Recent reviews suggest that oral nutritional supplementation (ONS) after hip fracture surgery may improve nutritional recovery, sarcopenia-related parameters, and functional outcomes, although the magnitude and consistency of benefit across clinical endpoints remain variable (13, 14). In parallel, emerging studies have highlighted the importance of actual postoperative dietary intake and protein provision for ambulation, body-composition recovery, and broader rehabilitation trajectories in older adults after hip fracture. These findings support the concept that early restoration of oral nutrient intake may be clinically meaningful in the immediate postoperative period, when routine food intake is often inadequate (15, 16). From a perioperative management perspective, current work also supports more structured nutritional pathways in elderly patients with hip fracture. Recent research has proposed dedicated perioperative nutritional management pathways for nutritionally vulnerable hip-fracture patients and has reinforced the need to integrate nutrition into multidisciplinary orthogeriatric care (14, 17). Moreover, frailty-oriented evidence indicates that poor nutritional status interacts with comorbidity burden and functional vulnerability, which may further compromise postoperative recovery. Nevertheless, the optimal timing of postoperative nutritional support, particularly early ONS after arthroplasty for femoral neck fracture, remains insufficiently defined in routine clinical practice (18–20).

Given these considerations, further evaluation of early postoperative nutritional intervention in malnourished older adults undergoing hip arthroplasty for femoral neck fracture is clinically warranted. This study aimed to determine whether early ONS is associated with more favorable perioperative nutrition-related indicators and postoperative recovery in this high-risk surgical population.

## 2 Methods

### 2.1 Study design

This retrospective study enrolled older patients with malnutrition who underwent hip arthroplasty for femoral neck fracture at our institution between January 2022 and December 2025. Eligible participants were consecutive patients aged 60 years or older with a confirmed diagnosis of femoral neck fracture based on clinical presentation and imaging findings, who received hemiarthroplasty or total hip arthroplasty during hospitalization. Patients were included if they had complete medical records, underwent standardized perioperative management, and had available data on nutritional indicators, postoperative recovery, and clinical outcomes. Patients were excluded if they had pathological fractures, multiple fractures or severe concomitant trauma, previous surgery on the affected hip, conservative treatment without arthroplasty, malignant tumors, severe hepatic or renal insufficiency, advanced gastrointestinal disease affecting enteral feeding tolerance or nutrient absorption, severe cognitive impairment or psychiatric disorders preventing reliable assessment, perioperative death within 24 h after surgery, or substantial missing clinical data. Patients were grouped according to whether ONS was initiated during the early postoperative period, and the effects of this intervention on nutritional status and postoperative recovery were subsequently evaluated. Informed consent was obtained from all participants. The study protocol was reviewed and approved by the ethics committee of our hospital. All procedures were conducted in accordance with relevant guidelines and regulations and complied with the Declaration of Helsinki. Patient confidentiality was strictly protected, and all identifiable information was removed before analysis.

### 2.2 Diagnostic criteria

The diagnosis of femoral neck fracture was established on the basis of clinical manifestations and imaging findings. Patients typically presented with hip pain, restricted movement of the affected limb, inability to bear weight, and, in some cases, limb shortening or external rotation deformity after trauma. The diagnosis was confirmed by preoperative imaging examinations, including plain radiography of the hip and pelvis, with computed tomography or magnetic resonance imaging performed when necessary to further clarify fracture location and characteristics.

Nutritional risk was screened at admission using the Nutritional Risk Screening 2002 (NRS-2002) tool. Patients with an NRS-2002 score of 3 or higher underwent further routine clinical nutritional assessment at our institution. In the present study, the NRS-2002 score was used for nutritional risk screening only. Malnutrition status at admission was identified according to documented diagnoses in the electronic medical records.

### 2.3 Grouping method

All patients received the same standard postoperative care pathway. Conventional postoperative care included routine intravenous fluid therapy, gradual advancement of the regular oral diet as tolerated, and routine postoperative rehabilitation according to the institutional care pathway. The only difference between groups was whether structured oral ONS was initiated within the first 24 h after surgery. Tube feeding was not used as part of the study intervention or group definition. Patients who received structured ONS within the first 24 postoperative hours were assigned to the early ONS group, whereas those who did not receive structured ONS during this period were assigned to the control group. In the early ONS group, supplementation was initiated within 24 h after surgery once oral intake was considered clinically feasible. The ONS used was a commercially available standard polymeric oral nutritional formula routinely used at our institution, with an energy density of approximately 1.5 kcal/mL. Each 200-mL serving provided approximately 300 kcal and 12 g of protein. The usual prescription was 200 mL twice daily, providing approximately 400 mL/day, 600 kcal/day, and 24 g of protein/day in addition to the regular postoperative diet. Supplementation was generally administered between meals and continued until discharge unless discontinued earlier because of intolerance, aspiration concern, or other clinical contraindications.

The initial volume could be reduced or divided into smaller portions in patients with reduced appetite or mild gastrointestinal discomfort. If nausea, vomiting, abdominal distension, diarrhea, or aspiration concern occurred, supplementation was temporarily withheld or reduced and then resumed according to clinical tolerance. Adherence was assessed according to the documented administered and consumed volumes. The daily completion rate was calculated as the actual consumed ONS volume divided by the prescribed ONS volume. Good adherence was defined as consumption of at least 80% of the prescribed ONS volume during the supplementation period.

Patients in the control group received the same standard postoperative care, including routine intravenous fluid therapy and gradual advancement of the regular oral diet as tolerated, but did not receive structured ONS within the first 24 h after surgery. Delayed nutritional supplementation could be initiated later when clinically indicated, according to the treating team’s assessment. No separate rehabilitation protocol was implemented according to nutritional group assignment.

### 2.4 Data collection

Clinical data were retrospectively collected from the electronic medical record system, nursing records, anesthesia records, laboratory information system, and inpatient follow-up documentation of our institution. Data extraction was performed independently by two investigators using a standardized case report form to ensure consistency and completeness, and key variables were cross-checked to improve data accuracy. The collected variables included demographic characteristics, baseline clinical information, surgery-related data, and nutrition-related indicators. Specifically, demographic characteristics included age, sex, body weight, body mass index, smoking history, and alcohol consumption history. Baseline clinical information included major comorbidities, such as hypertension, diabetes mellitus, coronary artery disease, chronic pulmonary disease, and cerebrovascular disease, as well as the American Society of Anesthesiologists physical status classification, preoperative anemia status, and preoperative nutritional risk. Surgery-related data included the type of surgical procedure, anesthesia method, operative time, intraoperative blood loss, and perioperative blood transfusion status. Nutrition-related indicators included serum albumin, prealbumin, hemoglobin, total protein, lymphocyte count, and the Nutritional Risk Screening 2002 score. When discrepancies were identified among different data sources, the original medical records were reviewed for verification. For missing data, variables with incomplete records were assessed during data cleaning, and cases with substantial missing information on key study variables were excluded from the final analysis. Additional postoperative variables within the first 24 h after surgery were extracted from anesthesia recovery records, nursing records, physician progress notes, and postoperative monitoring charts. These variables included recovery of consciousness/wakefulness, hemodynamic instability, persistent nausea or vomiting, marked abdominal distension or gastrointestinal intolerance, and swallowing safety or aspiration risk. Recovery of consciousness/wakefulness was defined as documented wakefulness with the ability to respond to verbal stimulation or follow simple instructions. Hemodynamic instability was defined as documented hypotension requiring active intervention, vasopressor use, repeated fluid resuscitation, or persistent concern regarding circulatory instability. Persistent nausea or vomiting, marked abdominal distension or gastrointestinal intolerance, and swallowing safety or aspiration risk were identified according to corresponding physician or nursing documentation. Patients were considered theoretically eligible for early ONS if they had adequate consciousness recovery and none of the other four limiting conditions within the first 24 postoperative hours. Information on ONS prescription, formula type, prescribed daily volume, actual consumed volume, duration of supplementation, gastrointestinal tolerance, and adherence was also extracted from physician orders, nursing administration records, nutrition records, and daily progress notes.

### 2.5 Outcome measures and time points for assessment

The outcome measures of this study were categorized as primary and secondary endpoints and were assessed during the index hospitalization at predefined time points or according to event occurrence. The primary outcome measures focused on postoperative nutritional and recovery-related outcomes, including changes in serum albumin, prealbumin, hemoglobin, total protein, and lymphocyte count after surgery, as well as length of hospital stay, time to first ambulation, and time to first flatus. These nutrition-related laboratory indicators were assessed relative to preoperative baseline values. The secondary outcome measures were the incidence of postoperative complications during the index hospitalization. Postoperative complications included pulmonary infection, wound-related complications, deep vein thrombosis, gastrointestinal intolerance, and urinary tract infection. In addition, the occurrence of any postoperative complication was evaluated as a composite secondary outcome. Baseline data were collected at admission before surgery. Nutrition-related laboratory indicators were assessed at predefined postoperative time points, including postoperative day 3 and at discharge. In this study, discharge was defined as release from the index hospitalization when the patient was judged by the treating team to be clinically stable and no longer in need of acute inpatient care. Variables assessed at discharge were based on the last available in-hospital clinical and laboratory measurements recorded before hospital discharge. Length of hospital stay was defined as the number of days from hospital admission to discharge during the index hospitalization. Time to first ambulation was defined as the time from surgery to the first documented out-of-bed ambulation, including standing and/or walking with assistance, as recorded in the medical or nursing records.

### 2.6 Statistical analysis

All statistical analyses were performed using IBM SPSS Statistics, version 30.0 (IBM Corp., Armonk, NY, USA). Analyses were based on the final complete-case cohort after exclusion of patients with substantial missing key data. Continuous variables were assessed for distributional characteristics and are presented as mean ± standard deviation (SD); between-group comparisons were performed using the independent-samples t test when appropriate. Categorical variables are presented as number (percentage) and were compared using the chi-square test or Fisher’s exact test, as appropriate. To evaluate the association between early oral ONS and postoperative outcomes, multivariable linear regression was used for continuous outcomes and multivariable logistic regression for binary outcomes. Covariates were selected *a priori* based on clinical relevance, prior literature, and potential confounding, rather than on univariable *p* values or stepwise procedures. Effect estimates are reported as regression coefficients (*β*) or odds ratios (ORs) with 95% confidence intervals (CIs). Subgroup analyses were conducted according to surgical procedure, ASA physical status, preoperative anemia, and nutritional risk. Sensitivity analyses included robust standard error models, log-transformed linear models for mildly right-skewed recovery outcomes, propensity score-adjusted models, and Firth penalized logistic regression for pulmonary infection. Covariate balance after propensity score adjustment was assessed using standardized mean differences, and multicollinearity was evaluated using variance inflation factors. A two-sided *p* value < 0.05 was considered statistically significant.

## 3 Results

### 3.1 Patient selection and enrollment

Between January 2022 and December 2025, 330 older patients with malnutrition who underwent hip arthroplasty for femoral neck fracture were identified. After exclusion of 36 patients, including those with pathological fracture (*n* = 6), multiple fractures or severe concomitant trauma (*n* = 8), severe missing clinical data (*n* = 11), advanced gastrointestinal disease affecting oral nutritional intake or absorption (*n* = 5), and perioperative death within 24 h after surgery (*n* = 6), a total of 294 patients were included in the final analysis. Of these, 118 patients received structured ONS within 24 h after surgery and were assigned to the early ONS group, whereas 176 patients who did not receive such supplementation during this period were assigned to the control group.

### 3.2 Intervention delivery and adherence

In the early ONS group, supplementation was initiated within 24 h after surgery following clinical assessment of oral intake feasibility (Supplementary Table S1). The usual prescribed dose was 200 mL twice daily, corresponding to approximately 600 kcal/day and 24 g of protein/day. The mean time from surgery to ONS initiation was 15.8 ± 4.6 h, and the mean duration of ONS use during hospitalization was 8.6 ± 2.7 days. The mean daily consumed volume was 356.4 ± 58.7 mL, corresponding to a mean completion rate of 89.1% ± 12.6%. Overall, 96 patients (81.4%) met the prespecified definition of good adherence, defined as consumption of at least 80% of the prescribed ONS volume. Gastrointestinal intolerance resulting in temporary dose reduction or interruption was documented in 4 patients (3.4%). No permanent discontinuation due to gastrointestinal intolerance was recorded, and all patients remained in the early ONS group for analysis.

### 3.3 Comparison of baseline demographic and clinical characteristics between the two groups

As shown in Table 1, baseline demographic and clinical characteristics were generally comparable between the early ONS group and the control group. The mean age was 79.3 ± 6.8 years in the early ONS group and 78.7 ± 7.1 years in the control group, with no significant difference (*p* = 0.467). The proportion of male patients was similar between groups (33.1% vs. 33.0%, *p* = 0.986). Body weight and body mass index also did not differ significantly between the two groups (*p* = 0.500 and *p* = 0.392, respectively). Smoking history and alcohol consumption history were comparable, with no significant between-group differences (*p* = 0.537 and *p* = 0.725, respectively). The prevalence of hypertension, diabetes mellitus, coronary artery disease, chronic pulmonary disease, and cerebrovascular disease was also similar between groups (all *p* > 0.05). In addition, the distribution of ASA physical status classification did not differ significantly between the early ONS group and the control group (*p* = 0.828). Although the proportion of patients with preoperative anemia was numerically higher in the early ONS group than in the control group (41.5% vs. 33.5%), the difference was not statistically significant (*p* = 0.163). Similarly, the preoperative NRS-2002 score was comparable between groups (*p* = 0.258).

**Table 1:** Baseline demographic and clinical characteristics of patients in the early ONS group and the control group.

Variable	Early ONS group (*n* = 118)	Control group (*n* = 176)	Test statistic	*p* value
Age, years	79.3 ± 6.8	78.7 ± 7.1	*t* = 0.73	0.467
Male sex, *n* (%)	39 (33.1)	58 (33.0)	*χ*^2^ = 0.00	0.986
Body weight, kg	57.4 ± 8.6	58.1 ± 8.9	*t* = −0.67	0.500
BMI, kg/m^2^	22.1 ± 2.9	22.4 ± 3.0	*t* = −0.86	0.392
Smoking history, *n* (%)	27 (22.9)	35 (19.9)	*χ*^2^ = 0.38	0.537
Alcohol consumption history, *n* (%)	22 (18.6)	30 (17.0)	*χ*^2^ = 0.12	0.725
Hypertension, *n* (%)	61 (51.7)	93 (52.8)	*χ*^2^ = 0.04	0.847
Diabetes mellitus, *n* (%)	34 (28.8)	44 (25.0)	*χ*^2^ = 0.53	0.468
Coronary artery disease, *n* (%)	28 (23.7)	36 (20.5)	*χ*^2^ = 0.44	0.505
Chronic pulmonary disease, *n* (%)	19 (16.1)	24 (13.6)	*χ*^2^ = 0.34	0.558
Cerebrovascular disease, *n* (%)	23 (19.5)	29 (16.5)	*χ*^2^ = 0.44	0.507
ASA physical status, *n* (%)			*χ*^2^ = 0.38	0.828
II	24 (20.3)	31 (17.6)		
III	71 (60.2)	108 (61.4)		
IV	23 (19.5)	37 (21.0)		
Preoperative anemia, *n* (%)	49 (41.5)	59 (33.5)	*χ*^2^ = 1.95	0.163
Preoperative NRS-2002 score	4.3 ± 0.7	4.2 ± 0.8	*t* = 1.13	0.258

ONS, oral nutritional supplementation; BMI, body mass index; ASA, American Society of Anesthesiologists; NRS-2002, Nutritional Risk Screening 2002.

### 3.4 Comparison of surgery-related variables and preoperative nutrition-related indicators between the two groups

With respect to perioperative characteristics, no significant differences were identified between the early ONS group and the control group. The distribution of surgical procedures was similar between groups, with hemiarthroplasty accounting for 61.0 and 63.6% of cases, respectively, and total hip arthroplasty accounting for 39.0 and 36.4%, respectively (*χ*^2^ = 0.21, *p* = 0.649). Anesthesia methods were also comparable, with spinal anesthesia used in 75.4% of patients in the early ONS group and 73.3% in the control group, and general anesthesia used in 24.6 and 26.7%, respectively (*χ*^2^ = 0.17, *p* = 0.683). Operative time, intraoperative blood loss, and perioperative blood transfusion rate did not differ significantly between groups (all *p* > 0.05). Preoperative nutrition-related indicators were likewise comparable. Serum albumin was 33.7 ± 3.5 g/L in the early ONS group and 33.3 ± 3.7 g/L in the control group (*p* = 0.349), and prealbumin was 156.8 ± 32.4 mg/L and 152.9 ± 34.1 mg/L, respectively (*p* = 0.323). No significant between-group differences were observed in hemoglobin, total protein, or lymphocyte count (all *p* > 0.05) (Table 2).

**Table 2:** Comparison of surgery-related variables and preoperative nutrition-related indicators between the early ONS group and the control group.

Variable	Early ONS group (*n* = 118)	Control group (*n* = 176)	Test statistic	*p* value
Surgery-related variables				
Surgical procedure, *n* (%)			*χ*^2^ = 0.21	0.649
Hemiarthroplasty	72 (61.0)	112 (63.6)		
Total hip arthroplasty	46 (39.0)	64 (36.4)		
Anesthesia method, *n* (%)			*χ*^2^ = 0.17	0.683
Spinal anesthesia	89 (75.4)	129 (73.3)		
General anesthesia	29 (24.6)	47 (26.7)		
Operative time, min	84.6 ± 15.8	86.1 ± 16.4	*t* = −0.79	0.433
Intraoperative blood loss, mL	221.5 ± 74.3	229.8 ± 78.6	*t* = −0.92	0.360
Perioperative blood transfusion, *n* (%)	38 (32.2)	49 (27.8)	*χ*^2^ = 0.65	0.422
Preoperative nutrition-related indicators				
Serum albumin, g/L	33.7 ± 3.5	33.3 ± 3.7	*t* = 0.94	0.349
Prealbumin, mg/L	156.8 ± 32.4	152.9 ± 34.1	*t* = 0.99	0.323
Hemoglobin, g/L	104.6 ± 13.8	102.7 ± 14.5	*t* = 1.13	0.258
Total protein, g/L	60.8 ± 5.9	60.1 ± 6.2	*t* = 0.98	0.330
Lymphocyte count, ×10^9/L	1.24 ± 0.36	1.19 ± 0.34	*t* = 1.19	0.234

ONS, oral nutritional supplementation; min, minutes; mL, milliliters.

### 3.5 Comparison of postoperative nutrition-related indicators between the two groups

On postoperative day 3, patients in the early ONS group had significantly higher levels of serum albumin, prealbumin, hemoglobin, total protein, and lymphocyte count than those in the control group (all *p* < 0.05). At discharge, these differences remained significant, with the early ONS group showing more favorable nutrition-related laboratory profiles than the control group. When changes from baseline were analyzed, both groups showed declines in most indicators on postoperative day 3, consistent with the acute postoperative metabolic response. However, the magnitude of decline in serum albumin, prealbumin, total protein, and lymphocyte count was significantly smaller in the early ONS group than in the control group (all *p* ≤ 0.030). The between-group difference in the decline in hemoglobin from baseline to postoperative day 3 was not statistically significant (*p* = 0.158). By discharge, changes in nutrition-related laboratory indicators remained more favorable in the early ONS group. Compared with baseline, patients in the early ONS group showed larger increases in serum albumin, prealbumin, total protein, and lymphocyte count than those in the control group (all *p* < 0.001). In addition, the reduction in hemoglobin at discharge was smaller in the early ONS group (*p* = 0.047). Overall, these findings indicate that ONS was associated with less pronounced decline in perioperative nutrition-related laboratory indicators and more favorable short-term postoperative laboratory profiles (Table 3).

**Table 3:** Comparison of postoperative nutrition-related indicators between the early ONS group and the control group.

Variable	Early ONS group (*n* = 118)	Control group (*n* = 176)	Test statistic	*p* value
Postoperative day 3				
Serum albumin, g/L	32.1 ± 3.4	30.8 ± 3.6	*t* = 3.14	0.002
Prealbumin, mg/L	149.6 ± 31.5	139.1 ± 32.8	*t* = 2.76	0.006
Hemoglobin, g/L	98.8 ± 12.9	95.4 ± 13.6	*t* = 2.17	0.031
Total protein, g/L	58.7 ± 5.6	57.2 ± 5.9	*t* = 2.20	0.028
Lymphocyte count, ×10^9/L	1.15 ± 0.31	1.03 ± 0.29	*t* = 3.34	<0.001
At discharge				
Serum albumin, g/L	34.5 ± 3.3	32.7 ± 3.5	*t* = 4.47	<0.001
Prealbumin, mg/L	177.4 ± 33.8	160.5 ± 34.2	*t* = 4.18	<0.001
Hemoglobin, g/L	103.2 ± 12.4	99.1 ± 13.1	*t* = 2.72	0.007
Total protein, g/L	62.8 ± 5.4	60.5 ± 5.7	*t* = 3.50	<0.001
Lymphocyte count, ×10^9/L	1.31 ± 0.33	1.16 ± 0.31	*t* = 3.91	<0.001
Change from baseline to postoperative day 3				
Δ Serum albumin, g/L	−1.6 ± 1.7	−2.5 ± 1.8	*t* = 4.35	<0.001
Δ Prealbumin, mg/L	−7.2 ± 16.5	−13.8 ± 17.6	*t* = 3.27	0.001
Δ Hemoglobin, g/L	−5.8 ± 8.7	−7.3 ± 9.2	*t* = 1.42	0.158
Δ Total protein, g/L	−2.1 ± 3.0	−2.9 ± 3.2	*t* = 2.18	0.030
Δ Lymphocyte count, ×10^9/L	−0.09 ± 0.15	−0.16 ± 0.16	*t* = 3.82	<0.001
Change from baseline to discharge				
Δ Serum albumin, g/L	0.8 ± 1.9	−0.6 ± 2.0	*t* = 6.06	<0.001
Δ Prealbumin, mg/L	20.6 ± 18.9	7.6 ± 19.8	*t* = 5.67	<0.001
*Δ* Hemoglobin, g/L	−1.4 ± 9.1	−3.6 ± 9.5	*t* = 2.00	0.047
Δ Total protein, g/L	2.0 ± 3.2	0.4 ± 3.4	*t* = 4.10	<0.001
Δ Lymphocyte count, ×10^9/L	0.07 ± 0.16	−0.03 ± 0.17	*t* = 5.12	<0.001

ONS, oral nutritional supplementation; Δ, change from baseline; g/L, grams per liter; mg/L, milligrams per liter.

### 3.6 Comparison of postoperative recovery outcomes between the two groups

Patients in the early ONS group achieved first ambulation significantly earlier than those in the control group (29.4 ± 8.6 h vs. 35.8 ± 10.4 h, t = −5.61, *p* < 0.001). Similarly, the time to first flatus was significantly shorter in the early ONS group than in the control group (41.7 ± 10.9 h vs. 49.6 ± 12.8 h, *t* = −5.48, *p* < 0.001), consistent with earlier return of postoperative bowel function. In addition, the length of hospital stay was significantly shorter in the early ONS group than in the control group (11.2 ± 2.8 days vs. 13.0 ± 3.4 days, *t* = −4.79, *p* < 0.001) (Table 4).

**Table 4:** Comparison of postoperative recovery outcomes between the early ONS group and the control group.

Variable	Early ONS group (*n* = 118)	Control group (*n* = 176)	Test statistic	*p* value
Time to first ambulation, h	29.4 ± 8.6	35.8 ± 10.4	*t* = −5.61	<0.001
Time to first flatus, h	41.7 ± 10.9	49.6 ± 12.8	*t* = −5.48	<0.001
Length of hospital stay, days	11.2 ± 2.8	13.0 ± 3.4	*t* = −4.79	<0.001

ONS, oral nutritional supplementation; h, hours.

### 3.7 Comparison of postoperative complications between the two groups

Pulmonary infection occurred in 5 patients (4.2%) in the early ONS group and 16 patients (9.1%) in the control group. Although the incidence was numerically lower in the early ONS group, the between-group difference did not reach statistical significance (*χ*^2^ = 2.35, *p* = 0.125). Likewise, no significant between-group differences were observed in incision-related complications (1.7% vs. 3.4%, *χ*^2^ = 0.75, *p* = 0.386), deep vein thrombosis (1.7% vs. 2.8%, *χ*^2^ = 0.39, *p* = 0.531), gastrointestinal intolerance (3.4% vs. 3.4%, *χ*^2^ = 0.00, *p* = 1.000), or urinary tract infection (3.4% vs. 6.8%, *χ*^2^ = 1.69, *p* = 0.194). Overall, 15 patients (12.7%) in the early ONS group and 31 patients (17.6%) in the control group developed at least one postoperative complication, with no statistically significant difference between groups (*χ*^2^ = 1.27, *p* = 0.260) (Table 5).

**Table 5:** Comparison of postoperative complications between the early ONS group and the control group.

Variable	Early ONS group (*n* = 118)	Control group (*n* = 176)	Test statistic	*p* value
Pulmonary infection, *n* (%)	5 (4.2)	16 (9.1)	*χ*^2^ = 2.35	0.125
Incision-related complications, *n* (%)	2 (1.7)	6 (3.4)	*χ*^2^ = 0.75	0.386
Deep vein thrombosis, *n* (%)	2 (1.7)	5 (2.8)	*χ*^2^ = 0.39	0.531
Gastrointestinal intolerance, *n* (%)	4 (3.4)	6 (3.4)	*χ*^2^ = 0.00	1.000
Urinary tract infection, *n* (%)	4 (3.4)	12 (6.8)	*χ*^2^ = 1.69	0.194
Any postoperative complication, *n* (%)	15 (12.7)	31 (17.6)	*χ*^2^ = 1.27	0.260

ONS, oral nutritional supplementation.

### 3.8 Multivariable adjusted analysis of the association between ONS and postoperative outcomes

To further evaluate the association between ONS and postoperative outcomes, multivariable regression analyses were performed after adjustment for potential confounding variables, including age, sex, body mass index, hypertension, diabetes mellitus, coronary artery disease, chronic pulmonary disease, cerebrovascular disease, ASA physical status, preoperative anemia, surgical procedure, anesthesia method, operative time, intraoperative blood loss, perioperative blood transfusion, and preoperative NRS-2002 score. In multivariable linear regression analyses, ONS was significantly associated with more favorable discharge nutrition-related laboratory indicators. Specifically, ONS was associated with higher serum albumin (*β* = 1.42, 95% CI: 0.66 to 2.18, *p* < 0.001), higher prealbumin (*β* = 13.58, 95% CI: 5.92 to 21.24, *p* < 0.001), higher total protein (*β* = 1.76, 95% CI: 0.54 to 2.98, *p* = 0.005), and higher lymphocyte count at discharge (*β* = 0.11, 95% CI: 0.04 to 0.18, *p* = 0.002). In addition, ONS was associated with shorter time to first ambulation (*β* = −5.38 h, 95% CI: −7.56 to −3.20, *p* < 0.001), shorter time to first flatus (*β* = −6.84 h, 95% CI: −9.53 to −4.15, *p* < 0.001), and shorter length of hospital stay (*β* = −1.29 days, 95% CI: −1.98 to −0.60, *p* < 0.001). In multivariable logistic regression analysis, ONS was associated with lower odds of pulmonary infection (OR = 0.46, 95% CI: 0.16 to 0.93, *p* = 0.041). However, because the number of pulmonary infection events was limited, this finding should be interpreted cautiously. The association between ONS and overall postoperative complications was not statistically significant after adjustment (OR = 0.71, 95% CI: 0.39 to 1.28, *p* = 0.254) (Table 6).

**Table 6:** Multivariable adjusted associations between ONS and postoperative outcomes.

Outcome variable	Adjusted effect estimate	95% CI	*p* value
Continuous outcomes (multivariable linear regression)			
Serum albumin at discharge, g/L	*β* = 1.42	0.66 to 2.18	<0.001
Prealbumin at discharge, mg/L	*β* = 13.58	5.92 to 21.24	<0.001
Total protein at discharge, g/L	*β* = 1.76	0.54 to 2.98	0.005
Lymphocyte count at discharge, ×10^9/L	*β* = 0.11	0.04 to 0.18	0.002
Time to first ambulation, h	*β* = −5.38	−7.56 to −3.20	<0.001
Time to first flatus, h	*β* = −6.84	−9.53 to −4.15	<0.001
Length of hospital stay, days	*β* = −1.29	−1.98 to −0.60	<0.001
Binary outcomes (multivariable logistic regression)			
Pulmonary infection	OR = 0.46	0.16 to 0.93	0.041
Any postoperative complication	OR = 0.71	0.39 to 1.28	0.254

ONS, oral nutritional supplementation; CI, confidence interval; *β*, regression coefficient; OR, odds ratio.

### 3.9 Subgroup and sensitivity analyses

Subgroup analyses were performed to examine whether the association between early ONS and postoperative recovery was consistent across clinically relevant strata. Length of hospital stay was selected as the representative recovery outcome. As shown in Supplementary Table S2, early ONS was associated with a shorter length of hospital stay in both the hemiarthroplasty and total hip arthroplasty subgroups, with no significant interaction according to surgical procedure. Similar directionally consistent associations were observed across ASA strata, preoperative anemia status, and nutritional risk categories, and no significant interaction was detected.

Sensitivity analyses were conducted to assess the robustness of the main findings. As shown in Supplementary Table S3, the associations of early ONS with more favorable discharge nutritional indicators, earlier ambulation, shorter hospital stay, and lower odds of pulmonary infection remained directionally consistent across the primary multivariable models, robust standard error models, and propensity score-adjusted models. The propensity score model included baseline demographic, clinical, nutritional, and perioperative variables selected *a priori*. Balance diagnostics (Supplementary Table S4) showed that, after propensity score adjustment, all absolute standardized mean differences were reduced to below 0.10, indicating adequate covariate balance.

To evaluate potential selection bias related to early postoperative readiness for oral intake, oral-intake feasibility variables during the first 24 h after surgery were compared between groups. As shown in Supplementary Table S5, no significant between-group differences were observed in recovery of consciousness/wakefulness, hemodynamic stability, nausea or vomiting, abdominal distension or gastrointestinal intolerance, swallowing safety or aspiration risk, or the proportion of patients meeting all five feasibility criteria, suggesting broadly comparable documented early postoperative feasibility for oral intake.

Because only 21 pulmonary infection events occurred, additional sensitivity analyses were performed for this outcome. As shown in Supplementary Table S6, the estimated association between early ONS and lower odds of pulmonary infection remained directionally consistent across the fully adjusted logistic regression, Firth penalized logistic regression, parsimonious logistic regression, and propensity score-adjusted models. However, given the limited number of events, this finding should be interpreted cautiously and considered exploratory.

Model diagnostics showed no substantial multicollinearity, with all variance inflation factors below 5 (Supplementary Table S7). In addition, because postoperative recovery outcomes showed mild right-skewness, sensitivity analyses using robust standard errors and log-transformed models were performed. As shown in Supplementary Table S8, the associations with shorter time to first ambulation, shorter time to first flatus, and shorter length of hospital stay remained directionally consistent, indicating that the findings were not materially driven by distributional assumptions. For transparency, the full multivariable regression models for the main outcomes are provided in Supplementary Table S9.

## 4 Discussion

The present study provides clinically relevant evidence that, in malnourished older adults undergoing hip arthroplasty for femoral neck fracture, early ONS initiated within 24 h after surgery was associated with less pronounced decline in perioperative nutrition-related laboratory indicators and more favorable early postoperative recovery. The novelty of this study lies in its focus on a particularly vulnerable and high-risk subgroup, namely older patients with both acute fragility fracture and pre-existing nutritional compromise, who are often underrepresented in perioperative nutrition research despite substantial metabolic stress and recovery burden. In addition to higher postoperative albumin, prealbumin, total protein, and lymphocyte count levels, our findings also showed associations with earlier ambulation, earlier return of bowel function, shorter hospitalization, and lower adjusted odds of pulmonary infection. From a clinical perspective, these results suggest that early postoperative ONS may represent a pragmatic, low-burden, and readily implementable component of perioperative management in this population. If incorporated into routine orthogeriatric care pathways, such an approach may support perioperative nutritional management and facilitate short-term in-hospital recovery in malnourished older patients after hip arthroplasty for femoral neck fracture.

These findings are biologically and clinically plausible in the context of geriatric hip fracture care. Older adults with hip fracture are particularly vulnerable to perioperative nutritional deterioration because acute trauma, surgical stress, immobility, inflammatory activation, anorexia, and multiple comorbidities often coexist. A recent systematic review and meta-analysis showed that malnutrition in older adults with hip fracture is associated with worse mobility and independence, as well as substantially higher short- and longer-term adverse outcome risks, underscoring the importance of perioperative nutritional management in this population (21). The 2025 ESPEN guideline on clinical nutrition in surgery emphasizes early restoration of oral feeding and early initiation of nutritional therapy when nutritional risk is present, while also linking perioperative nutrition with early mobilization and mitigation of stress-related catabolism (22). In this context, the present findings are broadly consistent with current perioperative nutrition principles and with the biological rationale that timely nutritional supplementation may help attenuate postoperative catabolic burden during the early recovery period (23).

A notable finding of the present study was the consistent pattern observed for nutrition-related laboratory indicators. Both groups showed an early postoperative decline in albumin, prealbumin, total protein, hemoglobin, and lymphocyte count, which is expected after hip fracture surgery because of surgical stress, inflammatory redistribution, perioperative hemodilution, and transient reductions in intake. However, declines in albumin, prealbumin, total protein, and lymphocyte count on postoperative day 3 were smaller in the early ONS group, and by discharge these indicators remained more favorable in this group. This pattern is concordant with recent hip-fracture nutrition studies. In a 2024 retrospective study of geriatric hip fracture patients undergoing total hip arthroplasty, postoperative ONS was associated with a smaller decline in serum protein and albumin and with higher albumin levels at follow-up (14). Similarly, a 2024 prospective observational study of a high-calorie, high-protein oral nutritional supplement with *β*-hydroxy-β-methylbutyrate reported more favorable nutritional status and biochemical profiles in malnourished or at-risk older hip-fracture patients (24). Taken together, these data suggest that early postoperative ONS may be associated with a less pronounced short-term decline in selected nutrition-related laboratory indicators after hip fracture surgery, although complete normalization during hospitalization may not be achievable. However, although the between-group differences in biochemical markers were statistically significant, their absolute magnitudes were relatively small and may not by themselves indicate clinically meaningful nutritional change. These laboratory findings should therefore be interpreted in the context of the accompanying recovery-related outcomes.

The observed associations with recovery-related endpoints are also clinically relevant. Earlier ambulation, earlier return of bowel function, and shorter hospitalization suggest that the association of early ONS may extend beyond laboratory indices and may also be reflected in more favorable early postoperative recovery during the index hospitalization. From a mechanistic perspective, timely nutrient provision may help attenuate perioperative energy-protein deficit, support muscle protein turnover, and maintain physiological reserve during the early postoperative period, which may in turn facilitate mobilization and tolerance of postoperative rehabilitation. The 2025 ESPEN surgical guideline explicitly positions nutrition and mobilization as interrelated components of perioperative care (22). In the aforementioned 2024 arthroplasty study, postoperative ONS was likewise associated with shorter hospital stay, and a 2025 randomized trial in older adults recovering from fragility hip fracture reported more favorable body-composition-related outcomes and good tolerability of an early oral supplement strategy (14, 25). Although direct comparisons across studies should be made cautiously because of differences in formulations, treatment duration, and outcome definitions, the overall direction of association is broadly consistent. Because direct standardized measures of functional recovery were not evaluated in the present study, these findings are best interpreted as relating to early postoperative recovery during hospitalization rather than comprehensive functional outcomes. Beyond mobility-related recovery, recent studies have also highlighted the broader perioperative vulnerability of older adults undergoing arthroplasty or hip fracture surgery, particularly with respect to neurocognitive outcomes. A recent trial protocol in elderly arthroplasty patients further underscored the clinical importance of postoperative delirium, pain, sleep, and quality of recovery as relevant perioperative endpoints, while a multicenter study in older adults with hip fracture developed and externally validated a dynamic nomogram for early postoperative neurocognitive disorder (26, 27). These reports further support the importance of multidimensional perioperative assessment in this vulnerable population.

The complication findings require a more cautious interpretation. In the crude comparison, pulmonary infection was numerically less frequent in the early ONS group, whereas the overall complication burden did not differ significantly between groups. After multivariable adjustment, early ONS remained associated with lower odds of pulmonary infection, whereas no significant association was observed for overall postoperative complications. This pattern is biologically plausible because respiratory infection in frail postoperative older adults may be influenced by nutritional status, immune competence, time to mobilization, and overall recovery trajectory. At the same time, the lack of a clear association with total complications suggests that any potential relationship between nutritional supplementation and postoperative morbidity may be outcome-specific and may be diluted when heterogeneous events are combined into a composite endpoint. This interpretation is broadly consistent with recent evidence indicating that ONS is associated with more favorable nutritional and selected clinical outcomes, although not all endpoints show uniform directional patterns across studies (28, 29). In view of the limited number of pulmonary infection events in the present cohort, this finding should be interpreted cautiously.

The adjusted, subgroup, and sensitivity analyses provide additional support for the consistency of the main findings. After adjustment for demographic characteristics, comorbidities, perioperative variables, preoperative anemia, and baseline nutritional risk, early ONS remained associated with more favorable discharge nutrition-related laboratory indicators and faster postoperative recovery. Subgroup analyses did not show significant interaction according to surgical procedure, ASA category, preoperative anemia status, or nutritional-risk stratum, suggesting that the direction of association was generally similar across clinically relevant patient subsets, although the estimates were less precise in the ASA IV subgroup. The consistency of these findings across alternative analytic approaches is relevant because nutritional support in routine hip-fracture care is frequently implemented non-randomly. In this context, a 2024 bi-national audit reported that fewer than half of older adults with hip fracture received ONS despite guideline-based recommendations, and that provision was related to patient-level clinical complexity (30). From a practical perspective, these findings support consideration of early ONS within standardized perioperative care for malnourished older adults undergoing hip arthroplasty for femoral neck fracture (31). ONS should, however, be considered within a broader orthogeriatric framework that includes early screening, individualized nutritional planning, pain control, mobilization, and complication prevention (32, 33).

Several limitations should be acknowledged. First, this was a single-center retrospective study with nonrandomized treatment allocation based on routine clinical practice, which limits causal inference and leaves the possibility of selection bias and residual confounding. Although multivariable adjustment, propensity score-adjusted analyses, subgroup analyses, and sensitivity analyses were performed, unmeasured confounders cannot be fully excluded. Second, although the two groups appeared broadly comparable with respect to documented early postoperative readiness for oral intake, differences in clinical stability and treatment selection may still have influenced the observed associations. Third, the nutrition-related laboratory indicators used in this study may be influenced by acute surgical stress, inflammation, fluid shifts, blood loss, transfusion, and hemodilution, and therefore do not in themselves establish true nutritional recovery, particularly in the early postoperative period. Fourth, total caloric and protein intake from all nutritional sources was not fully quantified. In addition, although all patients followed the same institutional postoperative care pathway and no separate rehabilitation protocol was implemented according to nutritional group assignment, variation in clinical status and care delivery within this shared pathway may still have influenced the observed recovery outcomes. Fifth, follow-up was limited to the index hospitalization, and longer-term functional, neurocognitive, and prognostic outcomes could not be evaluated. Accordingly, the present findings should be interpreted as supportive rather than definitive evidence. Future prospective multicenter studies, ideally randomized controlled trials, are needed to confirm these findings and to clarify the optimal timing, composition, duration, and target population for postoperative ONS.

## 5 Conclusion

Early ONS initiated within 24 h after hip arthroplasty was associated with less pronounced decline in perioperative nutrition-related laboratory indicators and faster postoperative recovery in malnourished older adults with femoral neck fracture. Lower adjusted odds of pulmonary infection were also observed, although this finding should be interpreted cautiously. Overall, these findings suggest that timely postoperative nutritional intervention may be a useful component of orthogeriatric perioperative care and support further prospective multicenter studies to define the optimal timing, composition, duration, and target population for postoperative nutritional supplementation.

## Data Availability

The raw data supporting the conclusions of this article will be made available by the authors, without undue reservation.
