# Supplementary material for: Early oral nutritional supplementation and postoperative recovery after hip arthroplasty in malnourished older adults with femoral neck fracture
Source: Front Nutr. 2026 Jul 22;13:1840514. doi: 10.3389/fnut.2026.1840514 (PMC13437495; doi:10.3389/fnut.2026.1840514)
Supplement: Supplementary file 1 [file Table_1.DOCX]

Supplementary Table S1. Delivery and adherence of oral nutritional supplementation in the early ONS group

| Variable | Early ONS group (n = 118) |
| --- | --- |
| Time from surgery to ONS initiation, h | 15.8 ± 4.6 |
| Initiation within 24 h after surgery, n (%) | 118 (100.0) |
| Prescribed ONS volume, mL/day | 400 |
| Prescribed energy, kcal/day | 600 |
| Prescribed protein, g/day | 24 |
| Duration of ONS during hospitalization, days | 8.6 ± 2.7 |
| Actual consumed ONS volume, mL/day | 356.4 ± 58.7 |
| Actual energy intake from ONS, kcal/day | 534.6 ± 88.1 |
| Actual protein intake from ONS, g/day | 21.4 ± 3.5 |
| Daily completion rate, % | 89.1 ± 12.6 |
| Good adherence, n (%) | 96 (81.4) |
| Temporary dose reduction/interruption due to gastrointestinal intolerance, n (%) | 4 (3.4) |

Abbreviations: ONS, oral nutritional supplementation; h, hours; mL, milliliters; kcal, kilocalories; g, grams.

Supplementary Table S2. Subgroup analyses of the association between early oral nutritional supplementation and favorable postoperative recovery outcomes

| Subgroup | n | Adjusted β for length of hospital stay, days | 95% CI | P value | Interaction P value |
| --- | --- | --- | --- | --- | --- |
| Surgical procedure |  |  |  |  | 0.412 |
| Hemiarthroplasty | 184 | -1.18 | -2.02 to -0.34 | 0.006 |  |
| Total hip arthroplasty | 110 | -1.46 | -2.58 to -0.34 | 0.011 |  |
| ASA physical status |  |  |  |  | 0.287 |
| ASA II–III | 234 | -1.36 | -2.10 to -0.62 | <0.001 |  |
| ASA IV | 60 | -0.88 | -2.01 to 0.25 | 0.127 |  |
| Preoperative anemia |  |  |  |  | 0.196 |
| Yes | 108 | -1.64 | -2.73 to -0.55 | 0.003 |  |
| No | 186 | -1.02 | -1.86 to -0.18 | 0.018 |  |
| Preoperative nutritional risk |  |  |  |  | 0.338 |
| NRS-2002 score 3–4 | 201 | -1.11 | -1.88 to -0.34 | 0.005 |  |
| NRS-2002 score ≥5 | 93 | -1.58 | -2.73 to -0.43 | 0.007 |  |

Abbreviations: ONS, oral nutritional supplementation; ASA, American Society of Anesthesiologists; NRS-2002, Nutritional Risk Screening 2002; CI, confidence interval; β, regression coefficient.

Supplementary Table S3. Sensitivity analyses of the association between early oral nutritional supplementation and major postoperative outcomes using different statistical models

| Outcome | Statistical model | Effect estimate | 95% CI | P value |
| --- | --- | --- | --- | --- |
| Serum albumin at discharge, g/L | Multivariable linear regression | β = 1.42 | 0.66 to 2.18 | <0.001 |
|  | Multivariable linear regression with robust standard errors | β = 1.39 | 0.61 to 2.17 | <0.001 |
|  | Propensity score-adjusted linear regression | β = 1.36 | 0.58 to 2.14 | 0.001 |
| Time to first ambulation, h | Multivariable linear regression | β = -5.38 | -7.56 to -3.20 | <0.001 |
|  | Multivariable linear regression with robust standard errors | β = -5.21 | -7.44 to -2.98 | <0.001 |
|  | Propensity score-adjusted linear regression | β = -4.97 | -7.18 to -2.76 | <0.001 |
| Length of hospital stay, days | Multivariable linear regression | β = -1.29 | -1.98 to -0.60 | <0.001 |
|  | Multivariable linear regression with robust standard errors | β = -1.24 | -1.95 to -0.53 | 0.001 |
|  | Propensity score-adjusted linear regression | β = -1.18 | -1.87 to -0.49 | 0.001 |
| Pulmonary infection | Multivariable logistic regression | OR = 0.46 | 0.16 to 0.93 | 0.041 |
|  | Firth penalized logistic regression | OR = 0.49 | 0.18 to 0.97 | 0.045 |
|  | Propensity score-adjusted logistic regression | OR = 0.52 | 0.21 to 0.99 | 0.048 |

Abbreviations: ONS, oral nutritional supplementation; CI, confidence interval; β, regression coefficient; OR, odds ratio; h, hours.

Supplementary Table S4. Balance diagnostics before and after propensity score adjustment

| Covariate | SMD before PS adjustment | SMD after PS adjustment |
| --- | --- | --- |
| Age | 0.086 | 0.031 |
| Sex | 0.002 | 0.006 |
| Body mass index | 0.101 | 0.028 |
| Hypertension | 0.022 | 0.014 |
| Diabetes mellitus | 0.086 | 0.032 |
| Coronary artery disease | 0.078 | 0.026 |
| Chronic pulmonary disease | 0.071 | 0.024 |
| Cerebrovascular disease | 0.078 | 0.029 |
| ASA physical status II | 0.068 | 0.023 |
| ASA physical status III | 0.025 | 0.018 |
| ASA physical status IV | 0.039 | 0.021 |
| Preoperative anemia | 0.165 | 0.047 |
| Surgical procedure | 0.053 | 0.019 |
| Anesthesia method | 0.047 | 0.017 |
| Operative time | 0.093 | 0.035 |
| Intraoperative blood loss | 0.108 | 0.041 |
| Perioperative blood transfusion | 0.096 | 0.038 |
| Preoperative NRS-2002 score | 0.133 | 0.044 |
| Maximum absolute SMD | 0.165 | 0.047 |

Abbreviations: ASA, American Society of Anesthesiologists; NRS-2002, Nutritional Risk Screening 2002; PS, propensity score; SMD, standardized mean difference.

Supplementary Table S5. Comparison of postoperative oral-intake feasibility variables during the first 24 hours after surgery

| Variable | Early oral nutritional supplementation group (n = 118) | Control group (n = 176) | P value |
| --- | --- | --- | --- |
| Adequate recovery of consciousness/wakefulness, n (%) | 113 (95.8) | 165 (93.8) | 0.451 |
| No documented hemodynamic instability, n (%) | 112 (94.9) | 164 (93.2) | 0.557 |
| No persistent nausea or vomiting, n (%) | 108 (91.5) | 155 (88.1) | 0.355 |
| No marked abdominal distension or gastrointestinal intolerance, n (%) | 110 (93.2) | 160 (90.9) | 0.492 |
| No swallowing safety concern or aspiration risk, n (%) | 113 (95.8) | 167 (94.9) | 0.72 |
| Met all five feasibility criteria, n (%) | 101 (85.6) | 146 (83.0) | 0.558 |

Supplementary Table S6. Sensitivity analyses for the association between early oral nutritional supplementation and pulmonary infection

| Model | Adjustment variables | Effect estimate | 95% CI | P value |
| --- | --- | --- | --- | --- |
| Crude model | None | OR = 0.44 | 0.16 to 1.24 | 0.13 |
| Primary fully adjusted logistic regression | Age, sex, BMI, hypertension, diabetes mellitus, coronary artery disease, chronic pulmonary disease, cerebrovascular disease, ASA physical status, preoperative anemia, surgical procedure, anesthesia method, operative time, intraoperative blood loss, perioperative blood transfusion, and preoperative NRS-2002 score | OR = 0.46 | 0.16 to 0.93 | 0.04 |
| Firth penalized logistic regression | Same covariates as the fully adjusted model | OR = 0.49 | 0.18 to 0.97 | 0.05 |
| Parsimonious logistic regression | Age, chronic pulmonary disease, ASA physical status, perioperative blood transfusion, and preoperative NRS-2002 score | OR = 0.48 | 0.19 to 0.95 | 0.04 |
| Propensity score-adjusted logistic regression | Propensity score adjustment | OR = 0.52 | 0.21 to 0.99 | 0.05 |

Abbreviations: ASA, American Society of Anesthesiologists; BMI, body mass index; CI, confidence interval; NRS-2002, Nutritional Risk Screening 2002; OR, odds ratio.

Supplementary Table S7. Collinearity diagnostics for covariates included in the adjusted models

| Covariate | VIF | Tolerance |
| --- | --- | --- |
| Age | 1.18 | 0.85 |
| Sex | 1.06 | 0.94 |
| Body mass index | 1.22 | 0.82 |
| Hypertension | 1.29 | 0.78 |
| Diabetes mellitus | 1.24 | 0.81 |
| Coronary artery disease | 1.31 | 0.76 |
| Chronic pulmonary disease | 1.18 | 0.85 |
| Cerebrovascular disease | 1.15 | 0.87 |
| ASA physical status III | 1.67 | 0.6 |
| ASA physical status IV | 1.74 | 0.57 |
| Preoperative anemia | 1.36 | 0.74 |
| Surgical procedure | 1.18 | 0.85 |
| Anesthesia method | 1.21 | 0.83 |
| Operative time | 1.41 | 0.71 |
| Intraoperative blood loss | 1.58 | 0.63 |
| Perioperative blood transfusion | 1.62 | 0.62 |
| Preoperative NRS-2002 score | 1.46 | 0.68 |

Abbreviations: VIF, variance inflation factor; ASA, American Society of Anesthesiologists; NRS-2002, Nutritional Risk Screening 2002.

Supplementary Table S8. Sensitivity analyses for postoperative recovery outcomes using robust and log-transformed models

| Outcome | Model | Effect estimate | 95% CI | P value |
| --- | --- | --- | --- | --- |
| Time to first ambulation, h | Primary linear regression | β = -5.38 h | -7.56 to -3.20 | <0.001 |
|  | Linear regression with robust standard errors | β = -5.21 h | -7.44 to -2.98 | <0.001 |
|  | Log-transformed linear regression | Ratio = 0.85 | 0.79 to 0.91 | <0.001 |
| Time to first flatus, h | Primary linear regression | β = -6.84 h | -9.53 to -4.15 | <0.001 |
|  | Linear regression with robust standard errors | β = -6.57 h | -9.31 to -3.83 | <0.001 |
|  | Log-transformed linear regression | Ratio = 0.86 | 0.81 to 0.92 | <0.001 |
| Length of hospital stay, days | Primary linear regression | β = -1.29 days | -1.98 to -0.60 | <0.001 |
|  | Linear regression with robust standard errors | β = -1.24 days | -1.95 to -0.53 | 0.001 |
|  | Log-transformed linear regression | Ratio = 0.90 | 0.85 to 0.96 | 0.001 |

Abbreviations: ASA, American Society of Anesthesiologists; NRS-2002, Nutritional Risk Screening 2002; CI, confidence interval; β, regression coefficient; h, hours.

Supplementary Table S9. Full multivariable regression models for the main outcomes

S9A. Full multivariable linear regression models for discharge nutritional indicators

| Variable | Serum albumin at discharge, β (95% CI) | P value | Prealbumin at discharge, β (95% CI) | P value | Total protein at discharge, β (95% CI) | P value | Lymphocyte count at discharge, β (95% CI) | P value |
| --- | --- | --- | --- | --- | --- | --- | --- | --- |
| Early oral nutritional supplementation | 1.42 (0.66 to 2.18) | <0.001 | 13.58 (5.92 to 21.24) | <0.001 | 1.76 (0.54 to 2.98) | 0.005 | 0.11 (0.04 to 0.18) | 0.002 |
| Age, per year | -0.03 (-0.08 to 0.02) | 0.214 | -0.42 (-0.91 to 0.07) | 0.091 | -0.04 (-0.12 to 0.04) | 0.318 | -0.003 (-0.008 to 0.002) | 0.236 |
| Male sex | 0.18 (-0.55 to 0.91) | 0.628 | 2.14 (-5.18 to 9.46) | 0.566 | 0.36 (-0.81 to 1.53) | 0.546 | 0.02 (-0.05 to 0.09) | 0.572 |
| Body mass index, per kg/m² | 0.09 (-0.03 to 0.21) | 0.143 | 1.18 (-0.05 to 2.41) | 0.06 | 0.14 (-0.05 to 0.33) | 0.148 | 0.006 (-0.005 to 0.017) | 0.276 |
| Hypertension | -0.22 (-0.92 to 0.48) | 0.537 | -1.76 (-8.83 to 5.31) | 0.625 | -0.33 (-1.46 to 0.80) | 0.567 | -0.01 (-0.08 to 0.06) | 0.776 |
| Diabetes mellitus | -0.46 (-1.20 to 0.28) | 0.222 | -3.98 (-11.42 to 3.46) | 0.293 | -0.61 (-1.80 to 0.58) | 0.315 | -0.03 (-0.10 to 0.04) | 0.397 |
| Coronary artery disease | -0.31 (-1.10 to 0.48) | 0.441 | -2.86 (-10.81 to 5.09) | 0.480 | -0.42 (-1.69 to 0.85) | 0.515 | -0.02 (-0.10 to 0.06) | 0.620 |
| Chronic pulmonary disease | -0.52 (-1.41 to 0.37) | 0.251 | -4.92 (-13.87 to 4.03) | 0.280 | -0.74 (-2.17 to 0.69) | 0.309 | -0.04 (-0.13 to 0.05) | 0.379 |
| Cerebrovascular disease | -0.39 (-1.20 to 0.42) | 0.345 | -3.41 (-11.56 to 4.74) | 0.411 | -0.55 (-1.85 to 0.75) | 0.405 | -0.03 (-0.11 to 0.05) | 0.461 |
| ASA physical status III | -0.48 (-1.34 to 0.38) | 0.274 | -4.58 (-13.24 to 4.08) | 0.299 | -0.68 (-2.06 to 0.70) | 0.333 | -0.04 (-0.12 to 0.04) | 0.331 |
| ASA physical status IV | -1.06 (-2.05 to -0.07) | 0.036 | -9.72 (-19.65 to 0.21) | 0.055 | -1.42 (-3.00 to 0.16) | 0.078 | -0.09 (-0.18 to 0.00) | 0.052 |
| Preoperative anemia | -0.58 (-1.28 to 0.12) | 0.104 | -5.35 (-12.39 to 1.69) | 0.136 | -0.83 (-1.95 to 0.29) | 0.146 | -0.05 (-0.12 to 0.02) | 0.159 |
| Total hip arthroplasty | 0.21 (-0.49 to 0.91) | 0.555 | 1.86 (-5.19 to 8.91) | 0.604 | 0.28 (-0.85 to 1.41) | 0.626 | 0.01 (-0.06 to 0.08) | 0.778 |
| General anesthesia | -0.44 (-1.21 to 0.33) | 0.262 | -4.13 (-11.88 to 3.62) | 0.296 | -0.59 (-1.83 to 0.65) | 0.350 | -0.03 (-0.10 to 0.04) | 0.397 |
| Operative time, per min | -0.01 (-0.03 to 0.01) | 0.278 | -0.10 (-0.28 to 0.08) | 0.276 | -0.01 (-0.04 to 0.02) | 0.513 | -0.001 (-0.003 to 0.001) | 0.317 |
| Intraoperative blood loss, per 50 mL | -0.16 (-0.34 to 0.02) | 0.082 | -1.48 (-3.32 to 0.36) | 0.115 | -0.23 (-0.52 to 0.06) | 0.119 | -0.01 (-0.03 to 0.01) | 0.254 |
| Perioperative blood transfusion | -0.71 (-1.46 to 0.04) | 0.064 | -6.62 (-14.15 to 0.91) | 0.085 | -1.05 (-2.25 to 0.15) | 0.086 | -0.06 (-0.13 to 0.01) | 0.091 |
| Preoperative NRS-2002 score | -0.44 (-0.89 to 0.01) | 0.056 | -4.26 (-8.77 to 0.25) | 0.064 | -0.62 (-1.34 to 0.10) | 0.091 | -0.04 (-0.08 to 0.00) | 0.069 |

Abbreviations: ASA, American Society of Anesthesiologists; NRS-2002, Nutritional Risk Screening 2002; CI, confidence interval; β, regression coefficient.

S9B. Full multivariable linear regression models for postoperative recovery outcomes

| Variable | Time to first ambulation, β (95% CI) | P value | Time to first flatus, β (95% CI) | P value | Length of hospital stay, β (95% CI) | P value |
| --- | --- | --- | --- | --- | --- | --- |
| Early oral nutritional supplementation | -5.38 (-7.56 to -3.20) | <0.001 | -6.84 (-9.53 to -4.15) | <0.001 | -1.29 (-1.98 to -0.60) | <0.001 |
| Age, per year | 0.15 (0.01 to 0.29) | 0.038 | 0.18 (0.01 to 0.35) | 0.041 | 0.04 (-0.01 to 0.09) | 0.108 |
| Male sex | -0.62 (-2.68 to 1.44) | 0.553 | -0.74 (-3.28 to 1.80) | 0.568 | -0.12 (-0.77 to 0.53) | 0.716 |
| Body mass index, per kg/m² | -0.18 (-0.52 to 0.16) | 0.299 | -0.21 (-0.63 to 0.21) | 0.327 | -0.04 (-0.15 to 0.07) | 0.476 |
| Hypertension | 0.74 (-1.23 to 2.71) | 0.459 | 0.86 (-1.57 to 3.29) | 0.487 | 0.21 (-0.41 to 0.83) | 0.506 |
| Diabetes mellitus | 1.46 (-0.62 to 3.54) | 0.169 | 1.82 (-0.74 to 4.38) | 0.164 | 0.42 (-0.23 to 1.07) | 0.205 |
| Coronary artery disease | 1.12 (-1.10 to 3.34) | 0.321 | 1.31 (-1.43 to 4.05) | 0.347 | 0.38 (-0.32 to 1.08) | 0.286 |
| Chronic pulmonary disease | 1.88 (-0.60 to 4.36) | 0.137 | 2.24 (-0.82 to 5.30) | 0.151 | 0.71 (-0.07 to 1.49) | 0.074 |
| Cerebrovascular disease | 1.74 (-0.52 to 4.00) | 0.131 | 2.15 (-0.64 to 4.94) | 0.13 | 0.56 (-0.15 to 1.27) | 0.122 |
| ASA physical status III | 1.62 (-0.78 to 4.02) | 0.185 | 2.04 (-0.92 to 5.00) | 0.176 | 0.48 (-0.27 to 1.23) | 0.209 |
| ASA physical status IV | 3.84 (1.05 to 6.63) | 0.007 | 4.76 (1.31 to 8.21) | 0.007 | 1.14 (0.27 to 2.01) | 0.01 |
| Preoperative anemia | 1.28 (-0.70 to 3.26) | 0.204 | 1.64 (-0.80 to 4.08) | 0.187 | 0.49 (-0.13 to 1.11) | 0.121 |
| Total hip arthroplasty | -0.84 (-2.82 to 1.14) | 0.405 | -0.96 (-3.40 to 1.48) | 0.440 | -0.18 (-0.80 to 0.44) | 0.568 |
| General anesthesia | 1.96 (-0.20 to 4.12) | 0.075 | 2.42 (-0.25 to 5.09) | 0.076 | 0.62 (-0.06 to 1.30) | 0.073 |
| Operative time, per min | 0.04 (-0.01 to 0.09) | 0.115 | 0.05 (-0.01 to 0.11) | 0.105 | 0.01 (0.00 to 0.03) | 0.091 |
| Intraoperative blood loss, per 50 mL | 0.42 (-0.08 to 0.92) | 0.099 | 0.51 (-0.11 to 1.13) | 0.107 | 0.16 (0.00 to 0.32) | 0.051 |
| Perioperative blood transfusion | 2.18 (0.08 to 4.28) | 0.042 | 2.56 (-0.04 to 5.16) | 0.054 | 0.82 (0.16 to 1.48) | 0.015 |
| Preoperative NRS-2002 score | 1.06 (-0.20 to 2.32) | 0.099 | 1.34 (-0.22 to 2.90) | 0.092 | 0.36 (-0.04 to 0.76) | 0.078 |

Abbreviations: ASA, American Society of Anesthesiologists; NRS-2002, Nutritional Risk Screening 2002; CI, confidence interval; β, regression coefficient; min, minutes.

S9C. Full multivariable logistic regression models for postoperative complications

| Variable | Pulmonary infection, OR (95% CI) | P value | Any postoperative complication, OR (95% CI) | P value |
| --- | --- | --- | --- | --- |
| Early oral nutritional supplementation | 0.46 (0.16 to 0.93) | 0.041 | 0.71 (0.39 to 1.28) | 0.254 |
| Age, per year | 1.04 (0.98 to 1.11) | 0.186 | 1.03 (0.99 to 1.08) | 0.152 |
| Male sex | 1.12 (0.42 to 2.98) | 0.821 | 1.08 (0.55 to 2.12) | 0.824 |
| Body mass index, per kg/m² | 0.94 (0.81 to 1.10) | 0.449 | 0.97 (0.87 to 1.08) | 0.575 |
| Hypertension | 1.18 (0.44 to 3.16) | 0.742 | 1.12 (0.58 to 2.17) | 0.737 |
| Diabetes mellitus | 1.42 (0.52 to 3.87) | 0.494 | 1.38 (0.69 to 2.77) | 0.364 |
| Coronary artery disease | 1.31 (0.45 to 3.82) | 0.621 | 1.26 (0.60 to 2.65) | 0.541 |
| Chronic pulmonary disease | 2.28 (0.82 to 6.33) | 0.113 | 1.74 (0.80 to 3.80) | 0.162 |
| Cerebrovascular disease | 1.56 (0.53 to 4.59) | 0.421 | 1.42 (0.66 to 3.05) | 0.369 |
| ASA physical status III | 1.62 (0.39 to 6.74) | 0.506 | 1.36 (0.55 to 3.35) | 0.504 |
| ASA physical status IV | 2.86 (0.69 to 11.85) | 0.147 | 2.08 (0.82 to 5.29) | 0.124 |
| Preoperative anemia | 1.41 (0.52 to 3.83) | 0.499 | 1.35 (0.68 to 2.68) | 0.39 |
| Total hip arthroplasty | 0.91 (0.32 to 2.58) | 0.859 | 0.94 (0.47 to 1.88) | 0.864 |
| General anesthesia | 1.74 (0.62 to 4.91) | 0.294 | 1.46 (0.70 to 3.04) | 0.313 |
| Operative time, per min | 1.01 (0.98 to 1.04) | 0.489 | 1.01 (0.99 to 1.03) | 0.318 |
| Intraoperative blood loss, per 50 mL | 1.17 (0.91 to 1.50) | 0.219 | 1.13 (0.95 to 1.34) | 0.165 |
| Perioperative blood transfusion | 1.82 (0.66 to 5.01) | 0.247 | 1.58 (0.77 to 3.24) | 0.213 |
| Preoperative NRS-2002 score | 1.31 (0.78 to 2.20) | 0.306 | 1.24 (0.87 to 1.77) | 0.235 |

Abbreviations: OR, odds ratio; CI, confidence interval; ASA, American Society of Anesthesiologists; NRS-2002, Nutritional Risk Screening 2002.
